# Relationship between neutrophils to HDL-C ratio and severity of coronary stenosis

**DOI:** 10.1186/s12872-020-01771-z

**Published:** 2021-03-06

**Authors:** Tuli Kou, Haorou Luo, Lixue Yin

**Affiliations:** 1 Southwest Medical University, Luzhou, China; 2 School of Medicine, University of Electronic Science and Technology of China, Chengdu, Sichuan China; 3 Sichuan Academy of Medical Sciences, Sichuan Provincial People’s Hospital, Chengdu, China

**Keywords:** Neutrophil, High-density lipoprotein cholesterol, Coronary stenosis, Coronary artery disease

## Abstract

**Background:**

Lipid and inflammatory molecules play a key role in the development of inflammation. Neutrophil counts are used as markers of inflammation duration, and HDL-C is used as an anti-atherosclerosis component. However, few studies have been found to integrate these two indicators to explore coronary stenosis. We suggested that neutrophil count as a marker of inflammation persistence and HDL-C as an anti-atherosclerotic component should be integrated into a single biomarker NHR to explore its correlation with CAD degree and predict the severity of coronary stenosis among CAD patients.

**Methods:**

We examined 404 eligible patients who underwent coronary angiography. Based on the results of coronary angiography, patients in CAD^+^ group (n = 155) were defined as those having angiographic coronary stenosis of at least 50% lumen reduction in at least one major coronary artery (including left anterior descending artery, left circumflex artery, left main coronary artery, right coronary artery). Patients with luminal stenosis but no more than 50% were defined as CAD^−^ group (n = 49), and patients without luminal stenosis (n = 200) were regarded as control group. The relationship between various serum markers and the severity of coronary stenosis was examined by Spearman correlation analysis. Logistic regression analysis was performed to identify the influencing factors of the severity of coronary artery disease.

**Results:**

The modified Gensini score was positively correlated with neutrophil HDL-C ratio and negatively correlated with albumin and HDL-C. Multiple regression analysis showed that neutrophil HDL-C ratio were significantly associated with CAD. Neutrophil HDL-C ratio is an independent predictor of CAD. The ROC analysis provided a cut-off value of 1.51 for neutrophil HDL-C ratio to predict CAD with 94.8% sensitivity and 0.024 Yoden index, and area under the ROC curve of 0.617 (95% CI 0.560–0.675, *P* < 0.001).

**Conclusion:**

Neutrophil HDL-C ratio is not only closely related to coronary artery stenosis, but also an independent predictor of severe coronary stenosis.

## Background

Coronary artery disease (CAD) is a catastrophic disease with high morbidity and mortality rates [1], causing adverse cardiac events in severe cases. CAD has the highest mortality rate in developed countries [2] and is now prevalent in developing countries as well; thus, it is becoming the primary cause of mortality worldwide [3]. Atherosclerosis plays a dominant role in the pathophysiological process of the disease, and studies have shown that CAD and atherosclerosis are closely associated with inflammation [1]. Meanwhile, lipid and inflammatory molecules play a key role in the development of inflammation. Current studies have shown that neutrophils contribute critically in the atherosclerotic process [4] and are markers of persistent inflammation as well as predictors of cardiovascular risk [5]. Moreover, high-density lipoprotein cholesterol (HDL-C) has been found to be closely related to coronary artery stenosis; it has the function of not only reverse cholesterol transport but also oxidation resistance and vascular endothelial function protection [6, 7],with its concentration being negatively correlated with the coronary heart disease risk [8]. Neutrophils and HDL-C are both important in the atherosclerotic process. However, few studies have been found to integrate these two indicators. Therefore, this study suggested that neutrophil count as a marker of inflammation persistence and HDL-C as an anti-atherosclerotic component should be integrated into a single biomarker NHR to explore its correlation with CAD degree and predict the severity of coronary stenosis among CAD patients [9].

## Methods

### Study population

This was a retrospective study consisting of 404 consecutive patients (174 males, 43.1%), who had undergone coronary angiography at the Sichuan Provincial People's Hospital from June 2018 to January 2020 due to CAD-related symptoms such as typical chest pain. All patients with coronary stent implantation, acute coronary Syndrome (ACS), coronary artery bypass surgery, liver and kidney disease, inflammatory disease, heart failure, blood diseases, rheumatic diseases, malignant tumors, alcohol use and antioxidant drugs (including statins, beta blockers, and aspirin) were excluded. Based on the results of coronary angiography, patients in CAD^+^ group (n = 155) were defined as those having angiographic coronary stenosis of at least 50% lumen reduction in at least one major coronary artery (including left anterior descending artery, left circumflex artery, left main coronary artery, right coronary artery).Patients with luminal stenosis but no more than 50% were defined as CAD^−^ group (n = 49), and patients without luminal stenosis (n = 200) were regarded as control group. Specifically, we did not include patients with diffuse coronary artery involvement but no stenosis. At the same time, when measuring the degree of obstuction on lumenogram, two experienced interventional cardiologists made a comprehensive judgment. The current study was in line with the principles of the Declaration of Helsinki.

### Clinical data and definitions

Height and weight were measured to calculate body mass index (BMI), which was counted as weight divided by height squared, weight in kilograms and height in meters. Collection of serum biomarkers such as uric acid, creatinine, triglycerides, bilirubin, total cholesterol, high-density lipoprotein cholesterol (HDL-C), albumin, low-density lipoprotein cholesterol (LDL-C), neutrophils, and eosinophils. Serum total antioxidant status of was calculated as: (0.63 × albumin concentration) + (1.53 × bilirubin concentration) + (1.02 × uric acid concentration), where the concentrations of uric acid, bilirubin and albumin were expressed in mmol/L. Neutrophil and HDL-C ratios were calculated. Hypertension was defined as blood pressure 140/90 mmHg or higher, or they were being given antihypertensive drugs. Those who had been diagnosed with hypertension in the past were all classified as having a history of hypertension. Patients were considered to have diabetes if they had a previous history of diabetes, or plasma glucose-lowering drugs use, including oral tablets and insulin. Current smokers were defined as those who smoked at least one cigarette a day for more than one year in a row; Long-term smokers who have quit for less than 6 months were still considered to have a smoking history.

### Gensini score system

The modified Gensini score (GS) system was used to measure CAD severity. These scores were expressed as the sum of the positional score of each lesion multiplied by the obstruction severity score, which emphasizes the severity of the disease. The calculation of the modified Gensini score system is shown in Table 1.

**Table 1:** Gensini score system

The degree of stenosis (%)	Score	Lesion location	Score
1–25	1	The second diagonal and other segments	0.5
26–50	2	Right coronary artery	1.0
51–75	4	The mid-distal region of the left circumflex artery	1.0
76–90	8	The distal left anterior descending artery, first diagonal branch	1.0
91–99	16	The mid-region of the left anterior descending artery	1.5
100	32	The proximal left anterior descending artery or proximal left circumflex artery	2.5
		The left main coronary artery	5.0

### Statistical analysis

All statistical analyses were performed using SPSS 25.0 for Mac. Kolmogorov–Smirnov test was used to verify that the continuous variable was normally distributed. The continuous variables of normal distribution were indicated as the mean ± standard deviation. Nonnormally distributed variables were represented as medians. Categorical variables were expressed in terms of frequency percentages and absolute numbers. Homogeneity of variance was evaluated by Levene’s test. If the data for continuous variables satisfied normal distribution and homogeneity of variance, a one-way analysis of variance was used, and the comparison among groups were performed with LSD method; Otherwise, the Kruskal–Wallis test was used to compare the differences of the non-conforming measurement data. Categorical variables were compared using the chi-squared. Correlation of Gensini score with serum biomarkers was analyzed by Pearson or Spearman’s correlation coefficient, as appropriate. Before further statistical analysis, Spearman’s correlation coefficients were first calculated to eliminate multicollinearity between or among variables. To determine the risk factors associated with CAD, multiple unconditional logistic regression models were performed as the primary analysis for the independent effects of each variable. Univariate logistic regression models were first performed to explore the crude association between the severity of coronary stenosis and each factor, including the ratio of neutrophils to high-density lipoprotein (NHR), sex, age, smoking, hypertension, albumin, creatinine, uric acid, HDL cholesterol, LDL cholesterol, and neutrophils individually. Factors that were significant in the univariate model at the p < 0.10 level were contained in the multivariate logistic regression model, so as to identify factors independently associated with the presence of coronary artery stenosis. Using the backward step-by-step approach, All statistically significant factors, i.e. P < 0.05 level, were contained in the final prediction model. A receiver operating characteristic (ROC) curve was presented. Regression coefficients and 95% confidence intervals (CIs) for risk factors significantly associated with CAD were calculated. All statistical tests were double-sided, and P value < 0.05 was considered statistically significant.

## Results

### Baseline characteristics

A total of 404 patients were included in our analysis. Demographic and clinical patient characteristics are presented in Table 2. The smoking incidence, hypertension incidence, creatinine, and neutrophil levels in CAD^+^ group were apparently higher than that in CAD^−^ group and control group (P < 0.05, respectively). Compared to the CAD^−^ group, the CAD^+^ group showed a significantly higher Gensini score (P < 0.05). Nevertheless, the levels of HDL-C and albumin in the CAD^+^ group were lower than those in the CAD^−^ group (P < 0.05). There were statistically significant differences in age and gender among the three groups (P < 0.05), and the average age of patients in the CAD^+^ group was higher than that in the CAD^−^ group (P < 0.001). The proportion of males in the CAD^+^ group was apparently higher than that in the CAD^−^ group and control group (P < 0.05, respectively). There were no significant differences in BMI, diabetes mellitus,cTAS,eGFR,bilirubin,total cholesterol, triglycerides,uric acid, and LDL-C, eosinophils among the three groups (P > 0.05, respectively).

**Table 2:** Clinical and laboratory characteristics of patients

Parameters	CAD^+^ group (n = 155)	CAD^−^ group (n = 49)	Control group (n = 200)	P
Age (year)	64.57 ± 10.57	64.22 ± 10.10	59.24 ± 10.69	< 0.001
Male [n (%)]	85 (54.8)	22 (44.9)	67 (33.5)	< 0.001
Body mass index (kg/m)	24.43 ± 3.21	24.19 ± 3.21	23.74 ± 4.13	0.212
Smoking [n (%)]	46 (29.7)	8 (16.3)	30 (15.0)	0.002
Hypertension [n (%)]	78 (50.3)	22 (44.9)	70 (35.0)	0.014
Diabetes mellitus [n (%)]	35 (22.6)	9 (18.4)	26 (13.0)	0.06
Creatinine (µm/L)	68.99 ± 26.60	65.90 ± 12.23	61.53 ± 14.08	0.009
Albumin (g/L)	41.76 ± 5.06	43.91 ± 4.04	42.77 ± 3.61	0.001
Total bilirubin (µm/L)	15.11 ± 6.81	16.24 ± 7.21	14.91 ± 6.17	0.403
Direct bilirubin (µm/L)	5.01 ± 3.81	5.00 ± 1.37	5.00 ± 1.41	0.102
Indirect bilirubin (µm/L)	10.26 ± 5.42	11.23 ± 6.10	9.97 ± 5.26	0.269
Total cholesterol (mmol/L)	4.92 ± 1.13	4.70 ± 0.95	4.74 ± 1.01	0.236
Triglyceride (mmol/L)	2.23 ± 1.74	1.96 ± 1.19	2.14 ± 1.75	0.429
Uric acid (µm/L)	328.74 ± 83.86	316.10 ± 93.58	318.50 ± 74.07	0.455
LDL-C (mmol/L)	2.82 ± 0.92	2.67 ± 0.82	2.62 ± 0.80	0.119
HDL-C (mmol/L)	1.28 ± 0.43	1.30 ± 0.29	1.35 ± 0.33	0.023
Neutrophils(× 10^9^/L)	4.67 ± 2.25	3.92 ± 1.63	4.08 ± 0.50	0.032
Eosinophil (× 10^9^/L)	0.94 ± 7.26	0.10 ± 0.09	0.11 ± 0.12	0.273
cTAS (mmol/L)	0.75 ± 0.10	0.76 ± 0.11	0.75 ± 0.09	0.592
Gensini score	18 (24.8–34.9)	3 (3.5–6.1)	0	< 0.001
NHR	3.99 ± 2.16	3.20 ± 1.75	3.24 ± 1.78	< 0.001

### Relationship between risk factors and the modified Gensini score

**Fig. 1:**
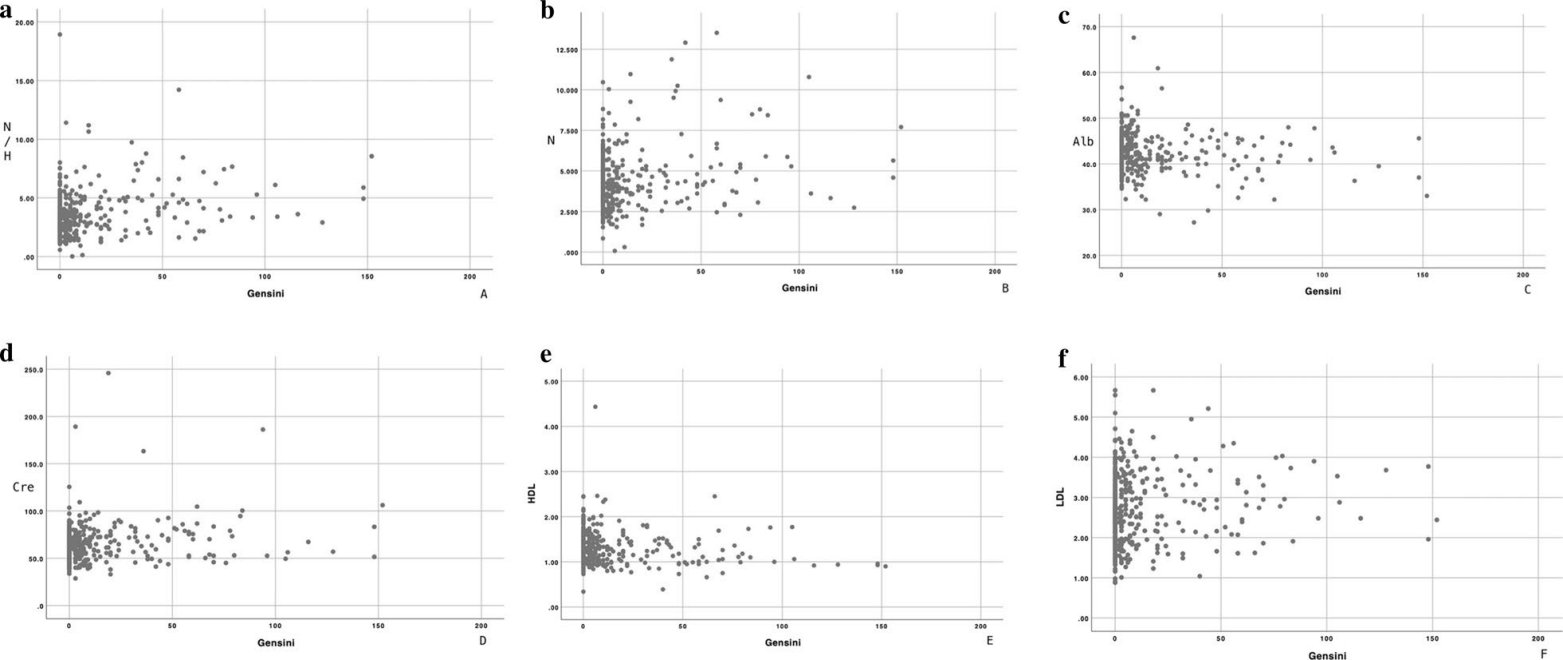
Relationship between serological indicators and Gensini score. **a** Relationship between Gensini score and NHR. **b** Relationship between Gensini score and N. **c** Relationship between Gensini score and Alb. **d** Relationship between Gensini score and Cre. **e** Relationship between Gensini score and HDL-C. **f** Relationship between Gensini score and LDL-C. NHR: the ratio of neutrophils to high-density lipoprotein; N: neutrophils; Alb: albumin; Cre: creatinine; HDL-C: high-density lipoprotein cholesterol; LDL-C: low-density lipoprotein cholesterol

To clarify the relationship between various risk factors and the modified Gensini score in patients with coronary atherosclerotic heart disease, Spearman rank correlation analysis was conducted. As shown in Table 3. The results showed that the Gensini score was positively correlated with NHR,neutrophils, creatinine, LDL-C, sex, age, cigarette smoking, hypertension, there was a negative correlation between Gensini score and HDL-C, as well as albumin. while Gensini score was not correlated with cTAS, eosinophil, uric acid, bilirubin, total cholesterol, triglyceride, and diabetes mellitus. The scatter diagram is shown in Fig. 1.

**Table 3:** Correlation coefficient between risk factors and Gensini score

Parameters	r	P
NHR and Gensini score	0.225	< 0.001
cTAS and Gensini score	0.013	0.788
Neutrophils and Gensini score	0.128	0.01
Eosinophil and Gensini score	-0.044	0.383
Uric acid and Gensini score	0.073	0.145
Creatinine and Gensini score	0.189	< 0.001
Albumin and Gensini score	− 0.13	0.009
Total bilirubin and Gensini score	0.03	0.551
Direct bilirubin and Gensini score	− 0.064	0.2
Indirect bilirubin and Gensini score 0.06	0.058	0.248
Total cholesterol and Gensini score	0.071	0.153
Triglyceride and Gensini score	0.058	0.245
LDL-C and Gensini score	0.113	0.023
HDL-C and Gensini score	− 0.189	< 0.001
Sex and Gensini score	0.143	0.004
Age and Gensini score	0.216	< 0.001
Cigarette smoking and Gensini score	0.117	0.019
Hypertension and Gensini score	0.104	0.036
Diabetes mellitus and Gensini score	0.049	0.323

### Univariate and multivariate logistic regression analyses

**Fig. 2:**
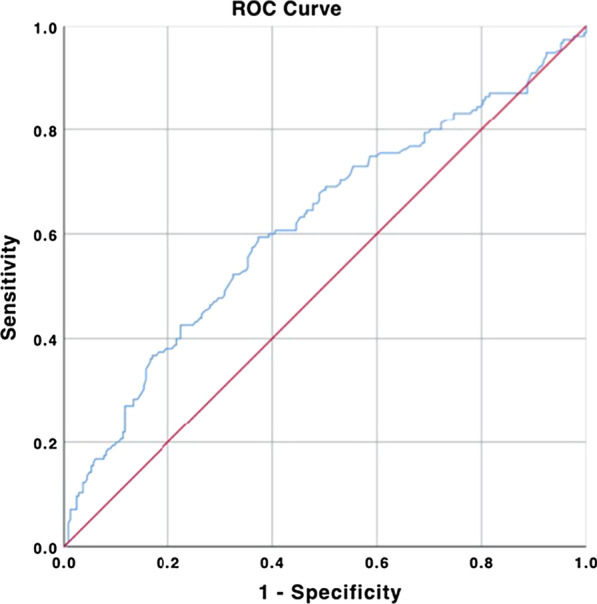
Receiver-operating characteristic curve analysis for NHR for prediction of CAD

To further explore the possible risk factors for coronary artery stenosis, univariate and multivariate logistic regression analyses were applied (Table 4). Univariate Logistic regression analyses showed that risk factors associated with CAD included NHR, smoking, hypertension, sex, age, creatinine, albumin, LDL-C, and central granulocyte (p < 0.05). Additionally, after adjusting for all covariates, multiple regression analyses showed that NHR, age, sex, albumin, and LDL-C were significantly correlated with CAD. Consequently, NHR was an independent predictor of CAD.The ROC analysis displayed that the critical value of NHR was 1.51, 94.8% of sensitivity and 0.024 of Yoden index could predict CAD, and the area under the ROC curve was 0.617 (95% CI 0.560–0.675, P < 0.001, Fig. 2).

**Table 4:** Logistic regression analysis of factors associated with the presence of CAD

Variables	Univariate		Multivariate	
	OR (95% CI)	P	OR (95% CI)	P
Creatinine	1.020 (1.008–1.033)	0.001	1.004 (0.992–1.016)	0.591
Albumin	0.921 (0.874–0.970)	0.002	0.944 (0.893–0.998)	0.043
NHR	1.232 (1.098–1.381)	< 0.001	1.163 (1.034–1.308)	0.012
Sex	2.183 (1.450–3.286)	< 0.001	2.664 (1.668–4.255)	< 0.001
Age	1.039 (1.019–1.060)	< 0.001	1.048 (1.025–1.071)	< 0.001
LDL	1.281 (1.013–1.620)	0.039	1.460 (1.130–1.886)	0.004
Neutrophils	1.204 (1.075–1.348)	0.001	1.010 (0.821–1.244)	0.82
Cigarette smoking	2.343 (1.439–3.817)	0.001	1.671 (0.900–3.104)	0.096
Hypertension	1.729 (1.151–2.596)	0.008	1.407 (0.894–2.214)	0.107

## Discussion

The results of this study showed that elevated NHR levels were closely correlated with the degree of coronary artery stenosis and that NHR was an independent predictor of CAD. Our study appears to be novel in that the use of NHR, which is a simple and readily available inflammatory marker, may help predict CAD and assess coronary artery stenosis.

Despite ongoing efforts to manage disease, such as strengthening medical care and advancements in invasive procedures, CAD remains the leading cause of death in patients with cardiovascular disease [10]. In patients presenting with chest pain, assessing the severity of coronary stenosis is critical in determining optimal treatment strategies and associated cardiovascular risk stratification [11]. The pathophysiological processes of CAD are complicated, which include altered lipid metabolism, inflammatory reaction, oxidative injury and other processes, in which various inflammatory factors are playing a central role [12, 13]. In this study, we hypothesised that NHR was a new indicator composed of inflammatory cells and lipid cholesterol that might reflect inflammation status and lipid metabolism more comprehensively than a single biological indicator. In the univariate and multivariate analyses, we investigated a number of factors associated with CAD, and the results showed that the Gensini score was positively correlated with NHR. After adjusting for confounding factors, NHR was found to be an independent predictor of CAD. Moreover, this study found that the NHR cutoff of 1.5 had a sensitivity of 94.8% in predicting artery disease.

Based on our analysis, we attempted to explain the association between higher NHR and severe coronary stenosis. Neutrophils are important components involved in immune inflammation [14, 15]. Previous studies have shown abundance of neutrophils in coronary artery lesions [16]. Recent evidences have suggested that elevated level of myeloperoxidase from neutrophils could contribute to coronary atherosclerosis [17]. Furthermore, neutrophils were associated with the complexity of coronary stenosis and were independent predictors of multiple complex stenoses [5]. Our results showed that the neutrophil level in the CAD^+^ group was higher than that in the coronary atherosclerosis group and control group, and the Gensini score was positively correlated with neutrophils, which was also consistent with the results of previous studies [18].

Dyslipidemia may account for 50% of the attributable risk in CAD population, so recent studies appear to pay more attention to the association between lipid-related biomarkers and coronary disease [19]. HDL-C, as a typical lipid-related biomarker, plays an important protective role in atherosclerosis and inflammation. Its mechanism of action was to transport excess cholesterol from peripheral tissues back to the liver for excretion. Meanwhile, it can inhibit the expression of endothelial cell adhesion molecules and prevent monocytes from accumulating into the arterial wall [20, 21]. More importantly, HDL-C inhibits neutrophil activation, diffusion and migration, which may be associated with lipid raft abundance [22].

Above all, because of the interaction of decreased HDL-C level and increased neutrophil count in CAD, an elevated NHR might be related to abnormal lipid metabolism and inflammatory activity. Furthermore, as regards the complex interaction between neutrophils and HDL-C, a comprehensive indicator of NHR may be more effective and reliable than a single indicator. In previous studies, NHR was calculated as a simple ratio of neutrophils and HDL-C, and related reports have assessed them as predictive markers of metabolic syndrome [23]. Another study showed a correlation between NHR and the extent of coronary artery disease [24]. However, that study focused on acute myocardial infarction, and our study was designed to directly discuss problems related to the severity of coronary artery lesions in CAD. In addition, we performed regression analysis of predictors of multiple indicators. Our large sample size was our advantage. Foremost, as a new predictor of CAD, NHR could be calculated from the complete blood count on admission, which is a fast and convenient method.

The Gensini score is an effective marker for assessing CAD severity. Coronary artery branches with varying severities have different weight coefficients, which can reflect disease severity more objectively [25, 26]. Previous studies have shown that Gensini scores were used as basis of grouping patients with severe CAD, and clear differences were found between the high score groups and other groups [27−29]. According to our results, NHR is positively correlated with Gensini scores. The combination of NHR and Gensini scores could help clinicians more effectively identify high-risk patients with coronary artery stenosis. In addition, relevant reports have suggested that cTAS is closely related to the severity of coronary stenosis [28, 29]. The expanded sample size test found that it was inconsistent with our results, possibly because the serum cTAS measured in the circulating blood may not be a true indicator of antioxidant concentrations in atherosclerotic plaques, and the equation for calculating cTAS in our study was neither very sensitive nor specific. Maryam et al. did not find that the cTAS level was an independent predictor of CAD, which was also consistent with our results [29].

In conclusion, we believe that NHR may be a widely used biomarker to predict CAD and assess the degree of coronary stenosis. However, our study had some limitations. First, this was a single-centre retrospective study, so there were limitations in persuasiveness. Furthermore, we did not explore the association of CRP, neopterin and other inflammatory markers with CAD. Finally, we did not explore the correlation between NHR and MACE. In future work, we will expand the sample size for further investigation.

## Conclusion

We found that NHR is not only closely related to coronary artery stenosis, but also an independent predictor of severe coronary artery stenosis and can be used as an independent indicator of the severity of coronary artery stenosis. Unlike many other biometrics, NHR is a relatively inexpensive and readily available marker that can provide effective value in predicting the severity of coronary stenosis (Additional file 1).

## Supplementary information

**Additional file 1: Figure 1.** Relationship between serological indicators and Gensini score.

## Data Availability

All data are freely available for scientific purpose. The data can be found from all listed authors.

## Abbreviations

NHR: Neutrophils to high density lipoprotein cholesterol ratio
CAD: Coronary artery disease
HDL-C: High density lipoprotein cholesterol
LDL-C: Low density lipoprotein cholesterol
ACS: Acute coronary syndrome
BMI: Body mass index
GS: Gensini score system
ROC: A receiver operating characteristic
CI: Confidence intervals
AMI: Acute myocardial infarction
LSD: Least significant difference
cTAS: The levels of calculated serum total antioxidant status
eGFR: Estimated glomerular filtration rate
